# Supplementary material for: Content-rich biological network constructed by mining PubMed abstracts
Source: BMC Bioinformatics. 2004 Oct 8;5:147. doi: 10.1186/1471-2105-5-147 (PMC528731; doi:10.1186/1471-2105-5-147)
Supplement: Additional File 2 — The original results of the above study (non-essential files are deleted to keep the file size under the limit set by BMC bioinformatics). [file 1471-2105-5-147-S2.bz2 › chilibotAdditionalFile2/dip05/287ID1326789E1115/html/MAP2K1_RAF1.html]

 


 **MAP2K1** and **RAF1** 
  
Found 1002 abstracts in PubMed, retrieved 05.  
 

 What does Google say? 
 PDF only 
| .edu only 

---

**Interactive relationship** (e.g. stimulation, inhibition, etc)

**Non-interactive relationship** (e.g. studied together, co-existance, homology, etc.)

- From this we conclude that a high affinity chitin oligosaccharide receptor system exists which links to the Raf  [ **RAF1** ] , MEK  [ **MAP2K1** ]  and ERK pathway in zebrafish cells.  Ref: 12881410 GlycobiologyGlycobiology,
- The RAF  [ **RAF1** ]  MEK  [ **MAP2K1** ]  ERK MAPK signal transduction cascade is an important mediator of a number of cellular fates including growth, proliferation and survival.  Ref: 12879021 Oncogene, 2003
- These events were accompanied by down regulation of the Raf 1  [ **RAF1** ]  MEK  [ **MAP2K1** ]  ERK pathway as well as diminished expression of Bcr Abl and cyclin D1, cleavage of p21 CIP1 and pRb, and induction of the stress related kinases JNK and p38 MAPK.  Ref: 12893773 BloodBlood, 2003
- Co treating PC 3 cells with quercetin significantly attenuated EGF and TGF alpha induced growth and phosphorylation of ErbB 2, ErbB 3, c Raf  [ **RAF1** ] , MAPK kinase 1 2 MEK1  [ **MAP2K1** ]  2, MAPK, Elk 1 and Akt 1.  Ref: 12888923 Int J Oncol, 2003
- Gastrin activated transcription through a Raf  [ **RAF1** ] , Mek  [ **MAP2K1** ]  and Erk dependent but Ras independent pathway.  Ref: 12891554 Gastroenterology, 2003
